# Importance of dietary supplementation of soluble and insoluble fibers to sows subjected to high ambient temperatures during late gestation and effects on lactation performance

**DOI:** 10.1016/j.aninu.2023.10.004

**Published:** 2023-11-14

**Authors:** Seung Min Oh, Abdolreza Hosseindoust, Sang Hun Ha, Jun Young Mun, Joseph Moturi, Habeeb Tajudeen, Yo Han Choi, Su Hyup Lee, Jin Soo Kim

**Affiliations:** a Gyeongbuk Livestock Research Institute, Yeongju 63052, Republic of Korea; b Department of Animal Industry Convergence, Kangwon National University, Chuncheon 24341, Republic of Korea; c Swine Division, National Institute of Animal Science, Rural Development Administration, Cheonan 31000, Republic of Korea; d Department of Swine Science, Korea National University of Agriculture and Fisheries, Jeonju 54874, Republic of Korea

**Keywords:** Soluble fiber, Insoluble fiber, Beet pulp, Wheat bran, Palm kernel, Metabolites

## Abstract

Heat stress adversely affects sows' performance, which can be improved by applying proper nutritional strategies. This study was conducted to investigate the interactive effects of dietary fiber levels and sources on sows' reproductive performance, metabolic response during gestation, and the carry-over influence on litter performance in the lactation period during heat stress (average room temperature of 27.1 °C). Fifty-four multiparous sows (Landrace × Yorkshire; initial body weight of 236.3 ± 16 kg; 2, 3 and 4 parities) at d 90 of gestation were assigned to a 2 × 3 factorial arrangement (9 sows/treatment), involving 2 dietary fiber levels (4.5% and 6% crude fiber) and 3 dietary fiber sources (wheat bran [WB], palm kernel meal [PK], and beet pulp [BP]). Sows fed the BP diet had highest (*P* < 0.01) feed intake and constipation index and lowest (*P* < 0.01) farrowing duration. Piglet weight (*P* = 0.041) and litter weight (*P* < 0.01) at weaning were higher in sows in the BP treatment compared to PK treatment. Sows in the BP treatment showed the greatest (*P* < 0.01) digestibility of crude protein and neutral detergent fiber. The fecal concentration of acetate was the lowest (*P* < 0.01) in the PK treatment. Total short-chain fatty acid production was increased in the WB and BP treatments compared with the PK. Sows in the BP treatment showed the lowest (*P* = 0.036) hair cortisol. The blood insulin concentration of sows was higher (*P* = 0.026) in the high fiber (6%) treatment compared with the low fiber (4.5%) treatment at 90 min and 120 min after the meal. The concentration of phthalic acid, succinic acid, phenylethylamine, hydrocinnamic acid, iron, linoleic acid, glycerol, ketone, and formamide were increased (*P* < 0.05) in the BP treatment compared with the WB. The BP treatment with high soluble fiber content improved the constipation index, farrowing duration, and litter performance, while high insoluble fibers increased sows comfort and reduced stress factors including respiratory rate and rectal temperature. Therefore, both soluble and insoluble sources of fiber are necessary to be added to the diet of gestating sows.

## 1 Introduction

Heat stress negatively affects seasonal fertility and litter performance in the swine industry (Brandt et al., 2022; He et al., 2019; Kim et al., 2019; Li et al., 2022). Previous studies suggested that when sows were exposed to temperature and humidity index (THI) values above 72–74, their reproductive performance, feed intake, and immune function was negatively impacted (Bjerg et al., 2020; Renaudeau et al., 2012). Therefore, the THI values measured in our study are considered to be in the range that could have significant impacts on sow health and productivity. When sows are subjected to heat stress, the reduced feed intake results in an imbalance of energy, body condition, and reproductive performance (Bjerg et al., 2020; Choi et al., 2017). Reduced feed intake and metabolic interaction are initial animal responses to mitigate metabolic heat increment (Bjerg et al., 2020; Choi et al., 2017; Thorsen et al., 2017). Both feed intake and fecal metabolic order can be affected in heat-stressed lactating sows. Furthermore, heat stress can decrease weaning to estrus intervals, farrowing rates, litter size, milk production, and weanling weight (Kim et al., 2019).

High dietary fiber (DF) intake in gestating sows has been associated with improved performance, welfare, and intestinal integrity (Jiang et al., 2019; Li et al., 2021; Serena et al., 2009). Reducing dietary fiber and protein intake is a common strategy to decrease heat increment and potentially manage the negative effects of high temperature (Choi et al., 2017; Schoenherr et al., 1989). There is limited knowledge on how the level and source of dietary fiber supplementation during heat stress can influence the performance. Fiber types are differentiated into soluble fibers (SF) and insoluble fibers (ISF) based on their physical and chemical characteristics. Soluble fibers contain pectin, β-glucan, gum, and hemicellulose, and the ISF contains cellulose, insoluble arabinoxylan, and lignin (Jørgensen et al., 2010; Taciak et al., 2010). Insoluble fibers' ability to bind with water increases fecal volume and promotes normal bowel movements, potentially reducing the incidence of constipation (Pearodwong et al., 2016). Additionally, SF contributes to decreased diarrhea incidence, improved gut health, increased water-holding capacity, and enhanced production of short-chain fatty acids (SCFA), promoting intestinal stability (Chen et al., 2021; Serena et al., 2009; Taciak et al., 2010; Wu et al., 2021a). Considering the advantages of fiber in improving intestinal growth in gestating sows, it is crucial to understand how variations in dietary fiber levels and sources can interactively mitigate the impacts of heat stress. In the current study, the diets were formulated based on fiber levels and sources to evaluate the effects on reproduction, litter growth, nutrient digestibility, stress status, and metabolic variations in the intestine.

## 2 Materials and methods

### 2.1 Animal ethics statement

This experiment was approved and conducted according to the guidelines of the Institutional Animal Care and Use Committee of Kangwon National University (KW-210503-6) and complied with the ARRIVE guidelines and complied with the ARRIVE guidelines.

### 2.2 Experimental design

**Fig. 1:**
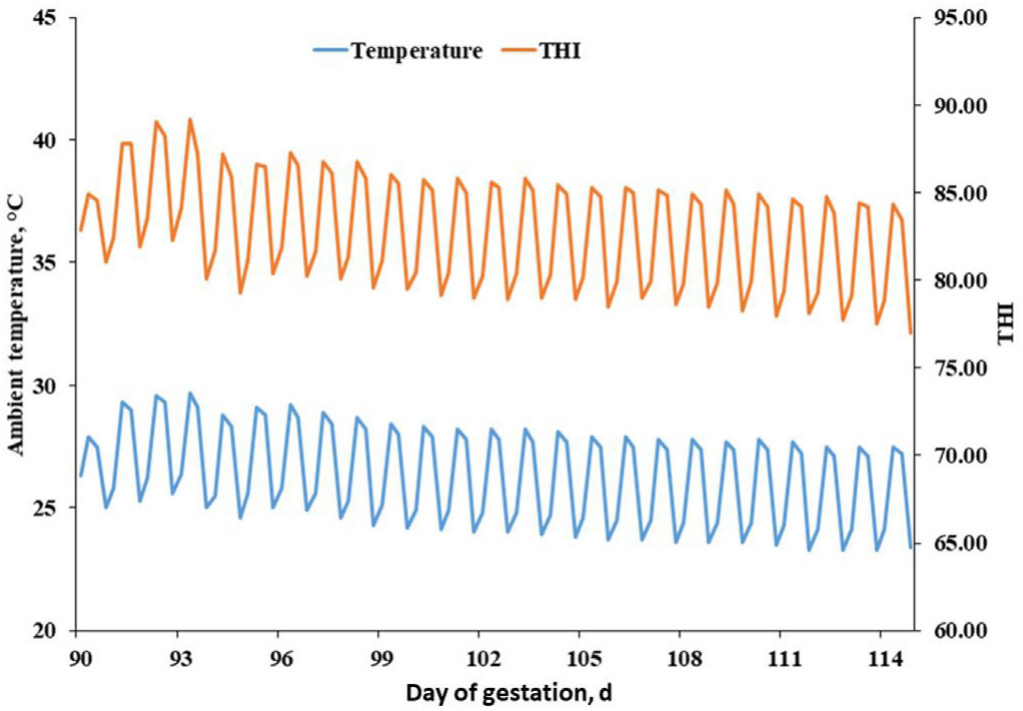
Ambient temperature and temperature-humidity index (THI) during experimental period.

The experiment was conducted in a Profarm farm in Gyeongsangnam-do Province July–August, 2021. All sows were subjected to heat stress during gestation and lactation. Despite all sows being exposed to heat stress during both gestation and lactation, the data collected revealed a relatively lower average THI during the lactation period. Fifty-four multiparous (Landrace × Yorkshire; initial body weight of 236.3 ± 16 kg) sows at d 90 of gestation and in their second (18 sows), third (18 sows), and fourth (18 sows) parities were selected. Sows were distributed between three blocks (parity two, three, and four) and six treatments (9 sows/treatment) as a 2 × 3 factorial arrangement with 2 crude fiber (CF) levels (4.5% and 6%) and 3 fiber sources as wheat bran (WB), palm kernel meal (PK) and beet pulp (BP). The gestation diets (Table 1) were prepared as recommended by NRC (2012). Sows were fed a gestation diet of 2.5 kg per day. The diet of lactating sows was according to NRC (2012) and contained 3300 kcal/kg of metabolizable energy (ME), 17.8% crude protein (CP), and 0.88% standardized ileal digestible lysine. Cross-fostering had been performed at the start of experiment. From the first day of lactation, the ration of the sows was gradually increased (1 kg/d) until the maximum (2 kg + 0.6 kg per a piglet) at 7 d post-partum. Throughout the lactation phase, each sow was individually housed in a stall (2.3 m × 0.75 m). The management protocol was performed according to Kim et al. (2018). In brief, sows were exposed to a boar, and two times artificial inseminations were conducted at the onset of estrus, and the Pharvision B-mode ultrasound machine was used for pregnancy detection on d 30 post-breeding (AV 2100 V; Ambisea Tech. Corp, Shenzhen, China). The individual stalls (2.05 m × 1.08 m) were used for gestating sows with fully slatted concrete flooring. On d 112 of gestation, each gestating sow was transferred to farrowing crates (2.14 m × 2.15 m). Feed and water were provided by a feeder and nipple drinker in each crate. Room humidity and temperature were checked at the sow's head level with 5 min intervals using a data logger (TM-305U, Tenmars Electronics Co., Taiwan, China). The accuracy and resolution of temperature loggers were ±0.4 and 0.01 °C, respectively. The accuracy and resolution of humidity loggers were ±3% and 0.2%, respectively. The following equation was used to calculate the THI. THI = temperature – [0.55 – (0.0055 × humidity)] × (temperature – 14.5). The room temperature and THI are shown in Fig. 1. Heating pads were installed on both sides of farrowing crates to maintain the temperature at 36 °C for piglets. Feed (NRC, 2012) and water were provided to lactating sows ad libitum with a drinker and feeder in each crate. The feeder troughs were checked and refilled three times daily when required. The leftover diet was collected and dried for feed intake calculation. Respiratory rates were counted by checking the rate of flank movement in a period of 60 s and calculated as breaths per min at 13:00 according to Nejad and Sung (2018) and Brandt et al. (2022).

**Table 1:** Ingredients and chemical composition of diets (%, DM basis).

Item	WB		PK		BP	
	4.5% CF	6.0% CF	4.5% CF	6.0% CF	4.5% CF	6.0% CF
Ingredients						
Corn	48.89	28.98	51.58	34.90	52.37	37.76
Wheat	4.00	4.00	4.00	4.00	4.00	4.00
SBM dehulled	7.79	1.32	7.74	–	8.46	3.22
Soy oil	3.76	7.53	2.95	5.65	3.35	4.76
WB	20.13	34.53	13.64	20.13	18.39	20.13
DDGS	12.00	20.00	12.00	22.80	5.00	20.00
PK	–	–	4.50	8.97	–	–
BP	–	–	–	–	3.30	6.60
Salt	0.50	0.50	0.50	0.05	0.50	0.50
Tricalcium phosphate	1.14	0.92	1.17	0.91	1.36	0.96
Limestone	1.05	1.25	1.02	1.24	0.79	1.06
DL-Methionine (98%)	0.02	0.03	0.04	0.07	0.06	0.03
L-Lysine (78.8%)	0.21	0.34	0.24	0.43	0.22	0.32
L-Tryptophan (10%)	0.11	0.17	0.21	0.39	0.20	0.24
L-Threonine (98.5%)	0.05	0.08	0.06	0.11	1.65	0.07
Choline-chloride (50%)	0.10	0.10	0.10	0.10	0.10	0.10
Vitamin premix^1^	0.10	0.10	0.10	0.10	0.10	0.10
Mineral premix^2^	0.10	0.10	0.10	0.10	0.10	0.10
Phytase	0.05	0.05	0.05	0.05	0.05	0.05
Total	100.00	100.00	100.00	100.00	100.00	100.00
Chemical composition^3^						
ME^4^, kcal/kg	3,140	3,140	3,140	3,140	3,140	3,140
CP	14.00	14.00	14.00	14.00	14.00	14.00
Calcium	0.82	0.82	0.82	0.82	0.82	0.82
Av. phosphorus	0.38	0.38	0.38	0.38	0.38	0.38
CF	4.50	6.00	4.50	6.00	4.50	6.00
Total dietary fiber^5^	15.01	22.81	14.82	22.50	14.49	20.18
ISF^5^	11.92	18.00	11.96	18.06	10.67	15.07
SF^5^	3.09	4.81	2.86	4.44	3.82	5.11
SID. Lys	0.58	0.58	0.58	0.58	0.58	0.58
SID. Met	0.26	0.22	0.28	0.25	0.27	0.22
SID. Met + Cys	0.42	0.42	0.42	0.42	0.42	0.42
SID. Thr	0.42	0.42	0.42	0.42	0.42	0.42
SID. Trp	0.13	0.13	0.13	0.13	0.13	0.13

WB = wheat bran; PK = palm kernel meal; BP = beet pulp; CF = crude fiber; SBM = soybean meal; DDGS = distillers dried grains with solubles; CP = crude protein; ISF = insoluble fibers; SF = soluble fibers; SID = standardized ileal digestibility.

1 The vitamin premix contained the following per kilogram of vitamin premix: 12,000,000 IU vitamin A, 2,400,000 IU vitamin D_3_, 132,000 IU vitamin E, 1,500 mg vitamin K_3_, 3,000 mg vitamin B_1_, 11,250 mg vitamin B_2_, 3,000 mg vitamin B_6_, 45 mg vitamin B_12_, 36,000 mg pantothenic acid, 30,000 mg niacin, 600 mg biotin, 4,000 mg folic acid.

2 The mineral premix contained the following per kilogram of mineral premix: 80,000 mg Fe, 170 mg Co, 8,500 mg Cu, 25,000 mg Mn, 95,000 mg Zn, 140 mg I, 150 mg Se.

3 The presented numbers are were calculated based on NRC (2012).

4 The metabolizable energy (ME) was calculated based on NRC (2012).

5 The presented numbers were calculated based on analyzed amounts.

### 2.3 Body weight and litter growth

Body weight and backfat thickness were measured on d 90 and 112 (pre-farrowing) of gestation and d 24 of lactation (weaning) according to Kim et al. (2019). Backfat thickness was measured at the 10th rib using an ultrasonic device (Agroscan A16, Angoulême, France). Litter performance, including the number of piglets born, born alive, body weight (kg) at birth and weaning, and number of piglets weaned were determined. Feed consumption (kg/d) and weaning-to-estrus interval (d) were also recorded. A total of 5 fecal samples were collected daily from each sow from the 110 d of gestation until the farrowing. The number of samples collected varied based on the farrowing time. The 0 to 5 scoring system was used to assess the consistency of each fecal sample: 0–no feces; 1–very hard and dry; 2–hard and dry; 3–normal consistency; 4–soft and loose; 5–very loose and watery. The fecal scores were determined for all of the fecal samples collected and divided by the total number of samples to obtain the average score.

### 2.4 Nutrient digestibility

From d 104 to 112 of gestation, chromic oxide (0.25%), an inert indigestible indicator, was added into the diet to evaluate the apparent total tract digestibility (ATTD) of dry matter (DM), gross energy (GE), CP, neutral detergent fiber (NDF), and acid detergent fiber (ADF). Fecal samples were collected at the last 4 d of gestation to measure the ATTD of DM, GE, CP, NDF, and ADF. The harvested samples were pooled within a pen and subjected to drying in a forced-air drying oven at 60 °C for 72 h, and then ground in a mill (Thomas Model 4 Wiley Mill, Thomas Scientific, Swedesboro, NJ, USA) using a 1-mm screen in order to determine chemical analysis. The samples were analyzed in triplicate with method number 930.15 for DM, 990.03 for CP, and 973.18 for ADF, 991.43 for total SF and ISF based on AOAC (2007). The GE of diet and feces was determined by a calorimeter bomb (Model 1261, Parr Instrument Co., Moline, IL), and the concentration of chromium (Fenton and Fenton, 1979) was measured using a spectrophotometer (Jasco V-650, Jasco Corp., Tokyo, Japan). The NDF determination was performed gravimetrically by mixing samples with neutral detergent, sodium sulfite, and amylase. The processed samples were filtrated with a glass filter (1.5-μm).

### 2.5 Short chain fatty acids determination

Fresh fecal samples were harvested by rectum massage on d 112 of gestation and kept at −80 °C, then transferred to the laboratory for analysis. Approximately 1 g of fecal sample was subjected to dilution with 2 mL of deionized water, then the sample was centrifuged at 10,000 × *g* at 4 °C for 20 min to obtain a supernatant (Mohammadigheisar et al., 2019). Afterward, a sample of 25% metaphosphoric acid solution was pooled in a 9:1 ratio and centrifuged at 3,000 × *g* for 10 min. The supernatant, aspirated with a syringe, was filtered using a 0.45-mm filter membrane. Gas chromatography (YL 6500 GC, Gyeonggi-do, Korea; TRB-G43 capillary column with the length of 30 m and inner diameter of 0.53 mm, and film thickness of 3 μm), outfitted with a flame ionization detector, and was used to analyze acetate, propionate, butyrate, and total SCFA. After 3 min, the column's temperature rose to 150 °C from a starting point of 70 °C. Each injection volume was 1 μL, and the temperature of the injector and detector was 250 °C.

### 2.6 Hair cortisol and blood glucose and insulin

The procedure for measuring hair cortisol has been previously described by Kim et al. (2020). In summary, sows' forehead hair was collected on d 90 and 110 of gestation. Aluminum foil was used to retain the gathered hair samples, and polypropylene tubes were used to dry them (HM Hyundai Micro Co., Korea). To remove contamination, the samples were washed three times with 50 mL isopropyl alcohol, and then they were dried for 7 d at room temperature (23 ± 1 °C). After drying, cortisol was extracted using methanol dilution and then evaluated using an ELISA kit (Cayman Chemical, Ann Arbor, MI; intra-assay CV = 9.05%, inter-assay CV = 9.6%) in accordance with the kit's instructions. A non-anticoagulant disposable tube was used to collect blood samples (10 mL) using an ear vein catheter from all the selected sows at gestational d 110 during the course of 4.5 h from 06:00 to 10:30 (Becton Dickinson, Franklin, NJ, USA). For blood glucose and insulin analyses, serum samples were separated by centrifugation (3,000 × *g* for 15 min at 4 °C). An automated chemistry analyzer employed a commercial kit (Fujifilm Corp., Saitama, Japan; intra-assay CV = 3.89%, inter-assay CV = 3.12%) to determine glucose (Fuji Dri-chem 3500i, Fujifilm Corp., Japan). In order to assess the blood insulin concentration, an ELISA kit (Endocrine Technologies Inc., New York, CA, USA; CV = 6.16%, inter-assay CV = 6.69%) and a spectrophotometer (Biolog MicroStation system, Hayward, CA, USA) was applied.

### 2.7 Sows' behavior

The Geovision GV-1240 video capture combo card (Geovision, Inc., Irvine, CA, USA) was utilized to capture and record sow behavior during gestation and lactation. Real-time observation of these behaviors was carried out using EZViewlog (Geovision, Inc., USA). From d 90 to 112 of gestation, as well as during farrowing, the behaviors of the sows were continuously recorded for 8 h (09:00–17:00) each day. The behaviors that were recorded, including ventral lying, lateral lying, sitting, standing, walking, drinking, eating, and farrowing.

### 2.8 Metabolomics analysis

Fresh fecal samples were harvested by rectum massage on d 112 of gestation. Metabolites concentrations of fecal samples were measured with GC-MS as described by He et al. (2019). Briefly, 500 mL of distilled water was used to dilute 100 mg of fresh fecal sample before it was vortexed for 60 s in 5-mL centrifuge tubes. As an internal quantitative reference, 1,000 mL of methanol was then added and vortexed for 30 s. After 30 min of incubation on ice, samples were held at 25 °C for 10 min using an ultrasonic machine, and then centrifuged at 3,075 × *g* for 15 min at 5 °C. After being dried, all of the supernatants were put in 2 mL centrifuge tubes. The dried samples were then combined with 60 L of methoxyamine solution in pyridine and vortexed (30 s) before being exposed to a 120-min 37 °C reaction period. After being mixed for 90 min at 37 °C with a reagent of 60 L trifluoroacetamide that contained 1% trimethylchlorosilane, the mixture was centrifuged for 15 min at 3,075 × *g* and 5 °C. The generated supernatant was transferred to a sample vial for Agilent 7890A/5975C GC-MS analysis (Agilent Technologies, Santa Clara, CA, USA).

### 2.9 Statistical analyses

The generalized linear model was used to perform the statistical analysis (SAS Inst. Inc. Cary, NC). Fiber levels, fiber sources, and parity number were fixed effects in the statistical model, while initial body weight was a covariate. Tukey multiple range tests were used to distinguish between means for each treatment in a 2 × 3 factorial arrangement. The experimental unit for each variable's investigation was an individual sow. Values of *P* ≤ 0.05 were regarded as significant. For the examination of metabolites, the gathered raw data were examined, and the metabolites were identified and normalized to (13C2)-myristic acid and stable isotope IS (http://srdata.nist.gov/gateway/). The software program SIMCA-P+ version 13.0 was used to carry out the statistical analysis (Umetrics, Umea, Sweden). As metabolites that may be compared between treatments, variables with variable importance projection (VIP) values of 1.0 and *P*-values of 0.05 were taken into consideration. The online tool (http://www.metaboanalyst.ca/faces/ModuleView.xhtml) was used to investigate how heat stress affected metabolic pathways and metabolite set enrichment analyses (He et al., 2019).

## 3 Results

### 3.1 Sow performance

The level and source of dietary fiber did not affect the body weight and backfat thickness during gestation (Table 2). The effects of fiber level on feed intake were insignificant. However, sows fed the BP diet had a higher feed intake compared to those fed the PK and WB diets (*P* = 0.002). The high fiber diet did not improve the farrowing duration of sows. However, dietary supplementation with BP significantly reduced the farrowing duration compared to the WB and PK diets (*P* < 0.01). The constipation index was not affected by dietary fiber level, but the BP diet showed a greater (*P* < 0.01) constipation index rather than the PK and WB diets. There was no difference in the weaning to estrus interval among the fiber level and source treatments. The fiber level and source showed no change in the total number of piglets born and weaned (Table 3). Sows in the BP treatment showed a higher piglet weight (*P* = 0.041) and litter weight (*P* < 0.01) compared with the PK treatment at weaning; however, no difference was observed between the WB and PK treatments.

**Table 2:** Effect of different fiber levels and sources on sows' performance.

Item	Fiber level		Fiber source			SEM	*P*-values		
	4.5%	6.0%	WB	PK	BP		Fiber level	Fiber source	Fiber level × Fiber source
Body weight, kg									
d 90	236.5	237.4	235.6	240.0	235.3	1.83	0.660	0.275	0.292
d 112	250.9	252.3	253.2	249.1	252.5	1.72	0.344	0.542	0.083
Weaning	215.8	216.7	217.8	212.9	218.1	2.14	0.838	0.531	0.760
Loss during lactation	35.11	35.61	35.39	36.27	34.41	0.82	0.719	0.442	0.516
Backfat thickness, mm									
d 90	21.3	21.2	21.7	20.4	21.6	0.41	0.805	0.395	0.455
d 112	21.2	21.9	21.6	21.8	21.2	0.41	0.368	0.826	0.457
Weaning	14.9	16.6	15.8	15.8	15.7	0.14	0.236	0.896	0.795
Loss during lactation	6.29	5.29	5.83	6.00	5.54	0.04	0.725	0.417	0.642
ADFI during lactation, kg/d	5.35	5.65	5.40^b^	5.21^b^	5.90^a^	0.08	0.051	0.002	0.574
Farrowing duration, h	4.72	4.58	4.69^b^	4.93^a^	4.34^c^	0.06	0.347	<0.001	0.621
Constipation index^1^	2.14	2.17	2.09^b^	1.93^b^	2.40^a^	0.04	0.763	<0.001	0.844
Weaning to estrus interval, d	5.32	5.42	5.40	5.63	5.08	0.12	0.675	0.174	0.985

WB = wheat bran; PK = palm kernel meal; BP = beet pulp; SEM = standard error of the mean; ADFI = average daily feed intake.

^a-c^ Means with different superscripts in the same row differ significantly (*P* < 0.05).

1 Constipation index: 0, absence of feces; 1, dry and pellet-shaped; 2, between dry and normal; 3, normal and soft, but firm and well formed; 4, between normal and wet, still formed, but not firm; and 5, very wet feces, unformed and liquid.

**Table 3:** Effect of different fiber levels and sources on litter performance.

Item	Fiber level		Fiber source			SEM	*P*-values		
	4.5%	6.0%	WB	PK	BP		Fiber level	Fiber source	Fiber level × Fiber source
Litter size									
Piglets born	12.4	13.0	12.9	12.6	12.7	0.17	0.197	0.696	0.737
Piglets weaned	10.7	11.1	10.9	10.6	11.1	0.14	0.159	0.348	0.844
Piglet weight, kg									
At birth	1.34	1.31	1.37	1.34	1.28	0.03	0.671	0.556	0.171
At weaning	5.95	6.07	6.01^ab^	5.89^b^	6.13^a^	0.07	0.439	0.041	0.503
Litter weight, kg									
At birth	15.4	15.5	15.9	15.4	15.0	0.41	0.865	0.646	0.381
At weaning	65.6	69.2	66.5^b^	63.1^b^	72.7^a^	1.27	0.164	0.010	0.568

WB = wheat bran; PK = palm kernel meal; BP = beet pulp; SEM = standard error of the mean.

^a,b^ Means with different superscripts in the same row differ significantly (*P* < 0.05).

### 3.2 Nutrient digestibility

Dry matter and GE digestibility were not influenced by dietary fiber level and source (Table 4). Sows in the BP treatment showed the greatest (*P* < 0.01) digestibility of CP. Moreover, a higher (*P* < 0.01) digestibility of CP was shown in the WB diet rather than in the PK diet. There was no difference in the digestibility of NDF and ADF among the fiber level treatments; however, a greater (*P* < 0.01) digestibility of NDF was observed in the BP compared with the PK and WB. In addition, the digestibility of NDF was increased (*P* < 0.01) in the PK treatment compared with the WB.

**Table 4:** Effect of dietary fiber levels and sources in gestation diets on apparent total digestibility (%).

Item	Fiber level		Fiber source			SEM	*P*-values		
	4.5%	6.0%	WB	PK	BP		Fiber level	Fiber source	Fiber level × Fiber source
DM	88.0	87.7	88.0	86.8	88.7	0.94	0.635	0.427	0.652
GE	88.6	87.9	88.0	87.5	89.3	0.61	0.566	0.173	0.437
CP	84.6	80.4	83.4^b^	76.5^c^	87.7^a^	0.72	0.178	<0.001	0.342
NDF	73.5	66.3	65.6^c^	69.4^b^	74.8^a^	1.07	0.073	<0.001	0.347
ADF	60.4	60.2	59.5^b^	61.1^a^	60.4^ab^	0.63	0.281	<0.001	0.494

WB = wheat bran; PK = palm kernel meal; BP = beet pulp; SEM = standard error of the mean; DM = dry matter; GE = gross energy; CP = crude protein; NDF = neutral detergent fiber; ADF = acid detergent fiber.

^a-c^Means with different superscripts in the same row differ significantly (*P* < 0.05).

### 3.3 Short-chain fatty acid production

Fiber level had no effect on the concentration of SCFA (Table 5); however, there was a tendency for higher fecal acetate (*P* = 0.088) and SCFA (*P* = 0.073) in sows fed high dietary fiber. The fecal concentration of acetate was decreased (*P* < 0.01) in sows fed the PK diet compared with the WB and BP. There was no difference in fecal propionate and butyrate concentrations. Total SCFA production was increased in the WB and BP treatments (*P* < 0.01) compared with the PK diet; however, no difference was shown between the WP and BP treatments.

**Table 5:** Effect of different fiber sources and levels on fecal SCFA concentration (μmol/g) in gestation sows.

Item	Fiber level		Fiber source			SEM	*P*-values		
	4.5%	6.0%	WB	PK	BP		Fiber level	Fiber source	Fiber level × Fiber source
Acetate	70.9	71.8	72.2^a^	69.2^b^	72.6^a^	0.25	0.088	<0.001	0.539
Propionate	17.6	17.8	17.6	17.2	18.3	0.20	0.566	0.095	0.840
Butyrate	7.47	7.53	7.51	7.54	7.47	0.04	0.440	0.798	0.515
Total SCFA	96.0	97.1	97.3^a^	93.9^b^	98.4^a^	0.31	0.073	<0.001	0.468

SCFA = short-chain fatty acids; WB = wheat bran; PK = palm kernel meal; BP = beet pulp; SEM = standard error of the mean.

^a,b^Means with different superscripts in the same row differ significantly (*P* < 0.05).

### 3.4 Stress indicators, plasma glucose and insulin

**Fig. 2:**
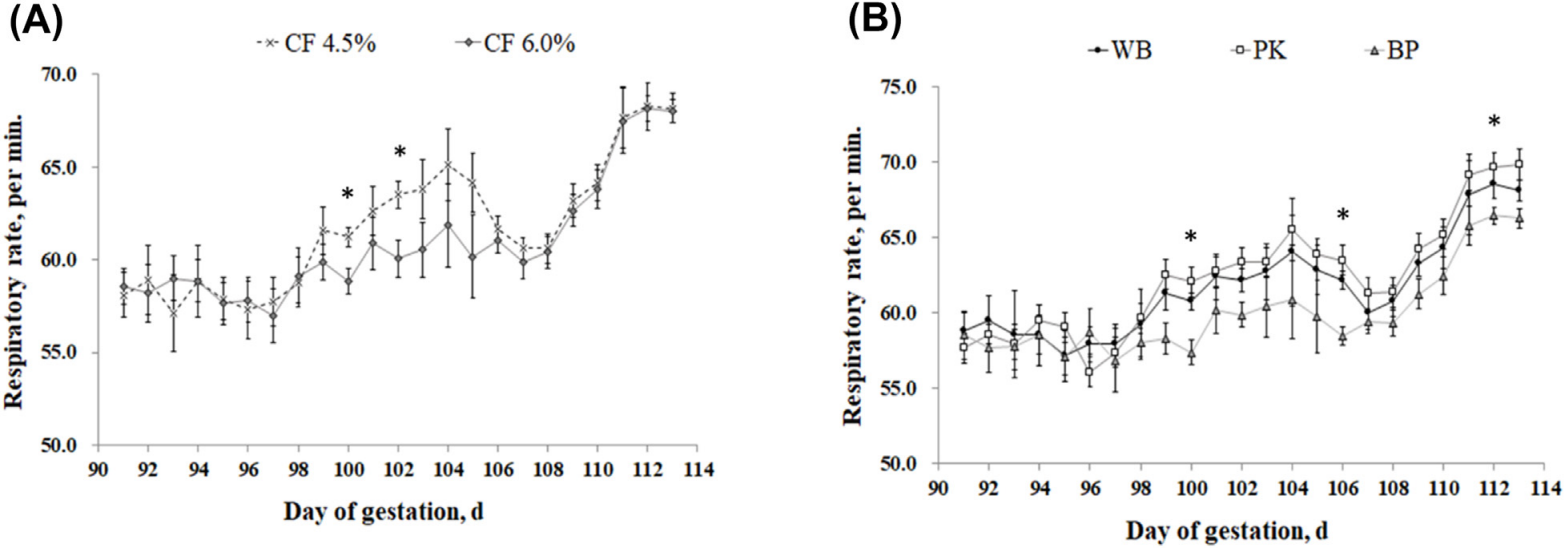
Effect of dietary fiber levels (A) and sources (B) on the respiratory rate of sows from d 90 to 114 of gestation at high ambient temperatures. Values represent means ± standard error from 54 sows. Asterisks (∗) indicate a statistical significance (*P* < 0.05). CF = crude fiber; WB = wheat bran; PK = palm kernel meal; BP = beet pulp.

**Fig. 3:**
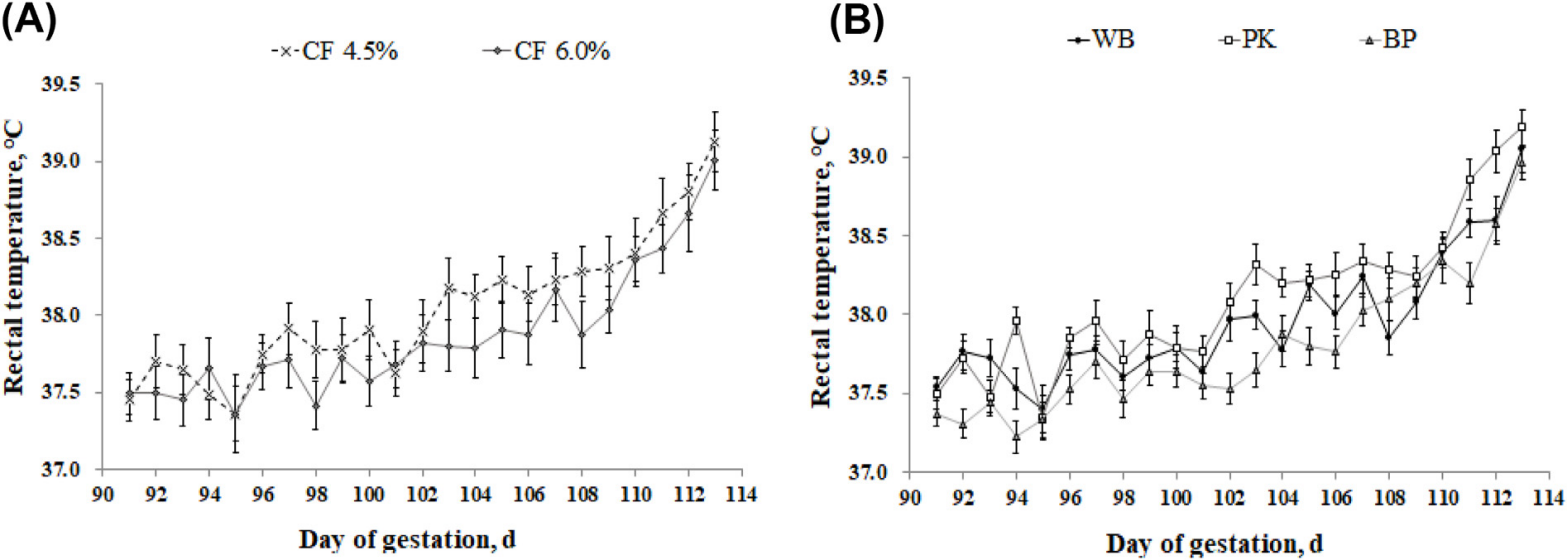
Effect of dietary fiber levels (a) and sources (b) of diets on the rectal temperature of 54 sows from d 90 to 114 of gestation at high ambient temperatures. CF = crude fiber; WB = wheat bran; PK = palm kernel meal; BP = beet pulp.

**Fig. 4:**
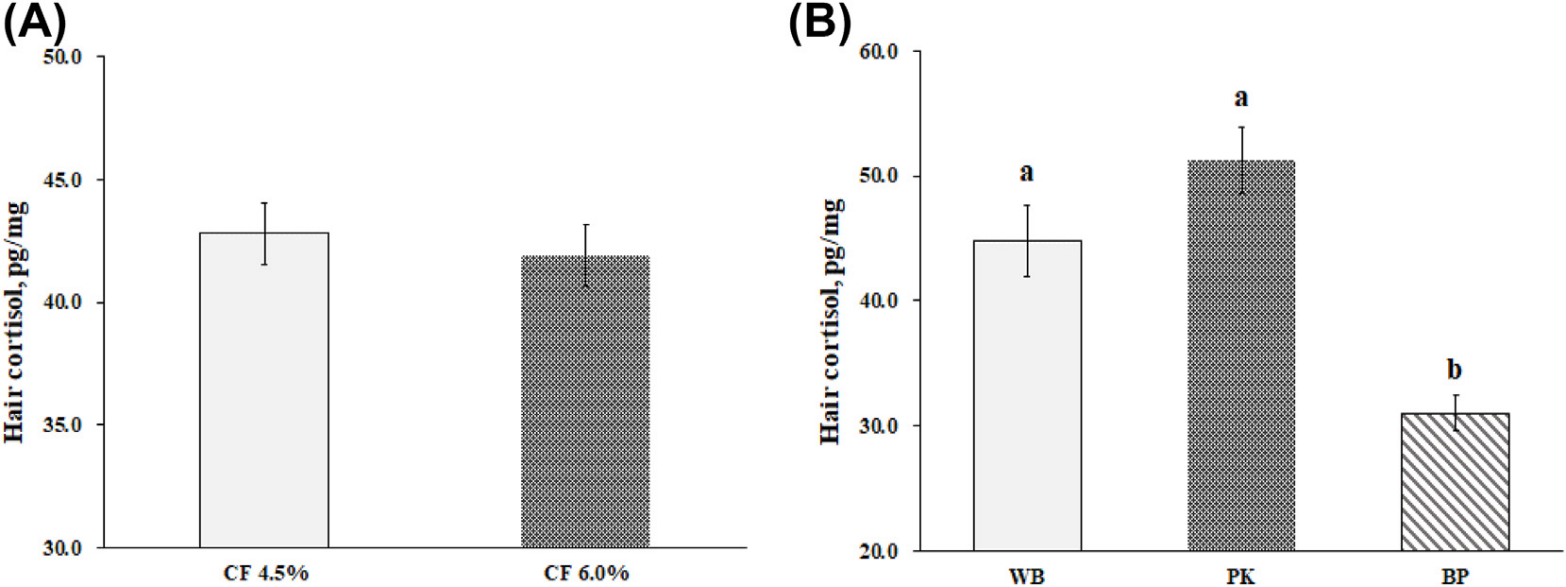
Effect of dietary fiber levels (a) and sources (b) of diets on the hair cortisol accumulation of sows from d 90 to 112 of gestation at high ambient temperatures. Values represent means ± standard error from 54 sows. ^a,b^ Means with different superscripts on the bar differ significantly (*P* < 0.05). CF = crude fiber; WB = wheat bran; PK = palm kernel meal; BP = beet pulp.

**Fig. 5:**
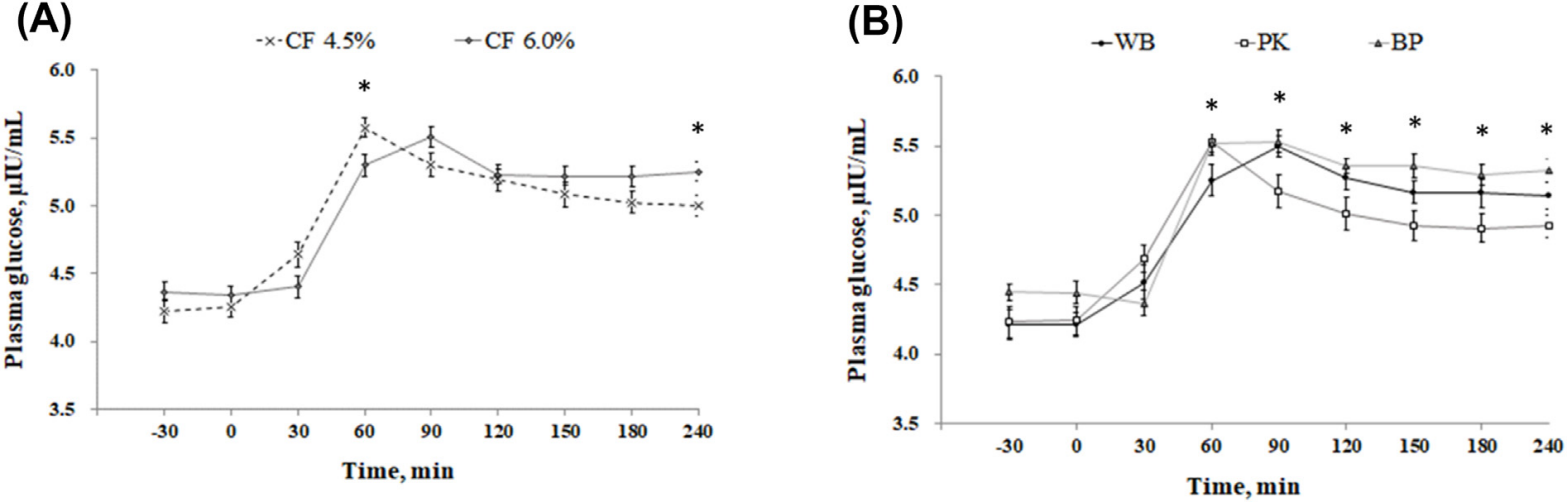
Effect of dietary fiber levels (a) and sources (b) of diets on the plasma glucose before or after meals of 54 gestating sows at high ambient temperatures (∗, *P* < 0.05). CF = crude fiber; WB = wheat bran; PK = palm kernel meal; BP = beet pulp.

**Fig. 6:**
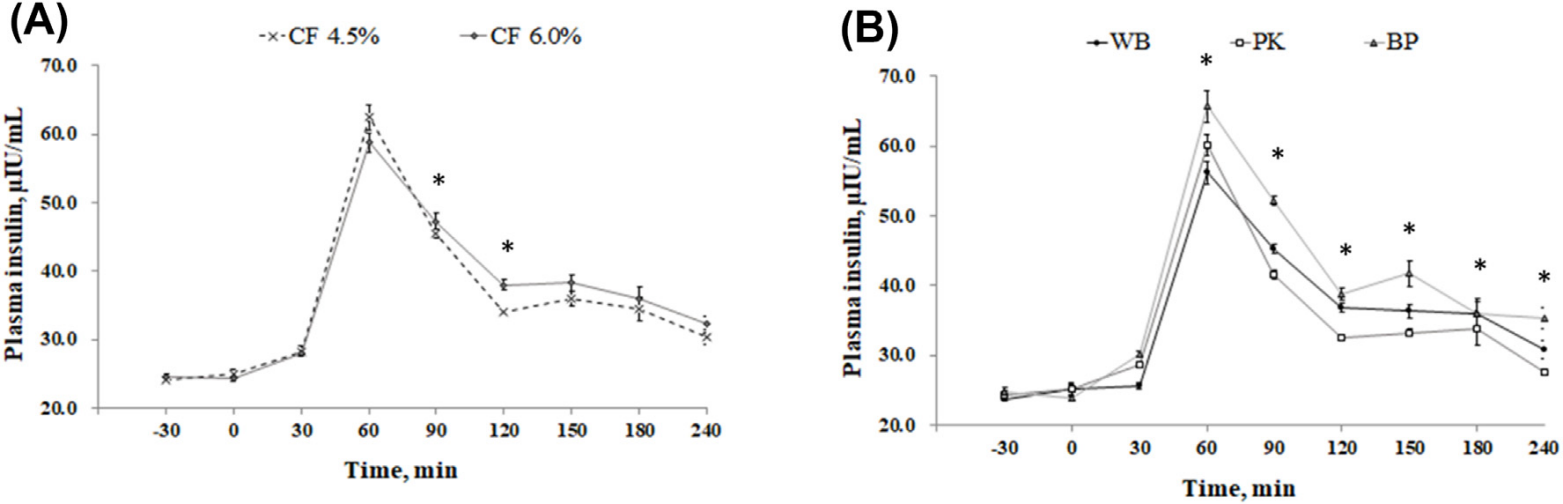
Effect of dietary fiber levels (a) and sources (b) of diets on the plasma insulin before or after meals of 54 gestating sows at high ambient temperatures (∗, *P* < 0.05). CF = crude fiber; WB = wheat bran; PK = palm kernel meal; BP = beet pulp.

Sows fed the high fiber diet showed a lower (*P* = 0.018) respiratory rate on d 100 and 102 compared with low fiber treatment; however, no difference was observed on d 90–98 and 104–114 (Fig. 2). There was no change in respiratory rate between the WB and PK diets; however, a lower respiratory (*P* = 0.011) rate was detected in sows fed BP compared with the WB and PK treatments at d 100, 106, and 112. Rectal temperature was not affected by dietary fiber level (Fig. 3); however, the BP treatment showed a lower rectal temperature (*P* = 0.020) compared with the PK treatment on d 92, 94, 96, 102, 103, 105, 106, 107, 111, and 112. Figure 4 illustrated the concentration of cortisol in hair. There was no variation of hair cortisol between fiber level treatments; however, sows in the BP treatment showed lower (*P* = 0.036) hair cortisol compared with the WB and PK treatments. The blood glucose level was higher (*P* = 0.029) in the low fiber treatment at 60 min after the meal and was lower (*P* = 0.018) at 240 min (Fig. 5). In addition, a greater (*P* < 0.01) blood glucose level was observed in the BP and WB treatments from 60 min after the meal to 240 min compared with the PK. The blood insulin concentration was higher (*P* < 0.026) in the high fiber treatment at 90 and 120 min after the meal (Fig. 6). A higher (*P* < 0.033) blood insulin level was observed in the BP treatment from 60 to 240 min after the meal compared with the WB and PK.

### 3.5 Behaviour

High dietary fiber levels increased lying (*P* < 0.01) and sitting (*P* < 0.01) behaviors and decreased (*P* < 0.01) standing behavior (Table 6). The drinking, licking, sniffing, bar-biting, and position change behaviors were not influenced by the level and source of dietary fiber. However, sows in the BP treatment showed the highest (*P* < 0.01) laying behavior among all treatments. Moreover, a lower (*P* < 0.01) standing and sham chewing behavior was shown in the BP treatment rather than in the WB and PK treatments.

**Table 6:** Effect of different fiber levels and sources on sows' behavior characteristics.

Item	Fiber level		Fiber source			SEM	*P*-values		
	4.5%	6.0%	WB	PK	BP		Fiber level	Fiber source	Fiber level × Fiber source
Lying, %	74.4	77.3	75.0^b^	73.0^b^	79.7^a^	0.60	<0.001	0.001	0.083
Sitting, %	15.9	16.6	15.8	17.2	15.8	0.60	<0.001	0.535	0.500
Standing, %	9.63	5.36	7.57^b^	9.67^a^	5.25^c^	0.35	<0.001	<0.001	0.061
Drinking, %	4.23	2.97	3.55	4.10	3.15	0.37	0.090	0.572	0.973
Sham chewing times	9.60	9.10	10.13^a^	11.65^a^	6.28^b^	0.61	0.684	0.002	0.722
Licking and sniffing times	2.83	2.60	2.65	3.00	2.50	0.34	0.732	0.827	0.969
Bar-biting times	4.83	4.00	4.30	5.25	3.70	0.33	0.217	0.170	0.859
Position change times	5.60	5.80	5.80	6.75	4.55	0.40	0.806	0.093	0.908

WB = wheat bran; PK = palm kernel meal; BP = beet pulp; SEM = standard error of the mean.

^a-c^ Means with different superscripts in the same row differ significantly (P < 0.05).

### 3.6 Metabolomics

**Fig. 7:**
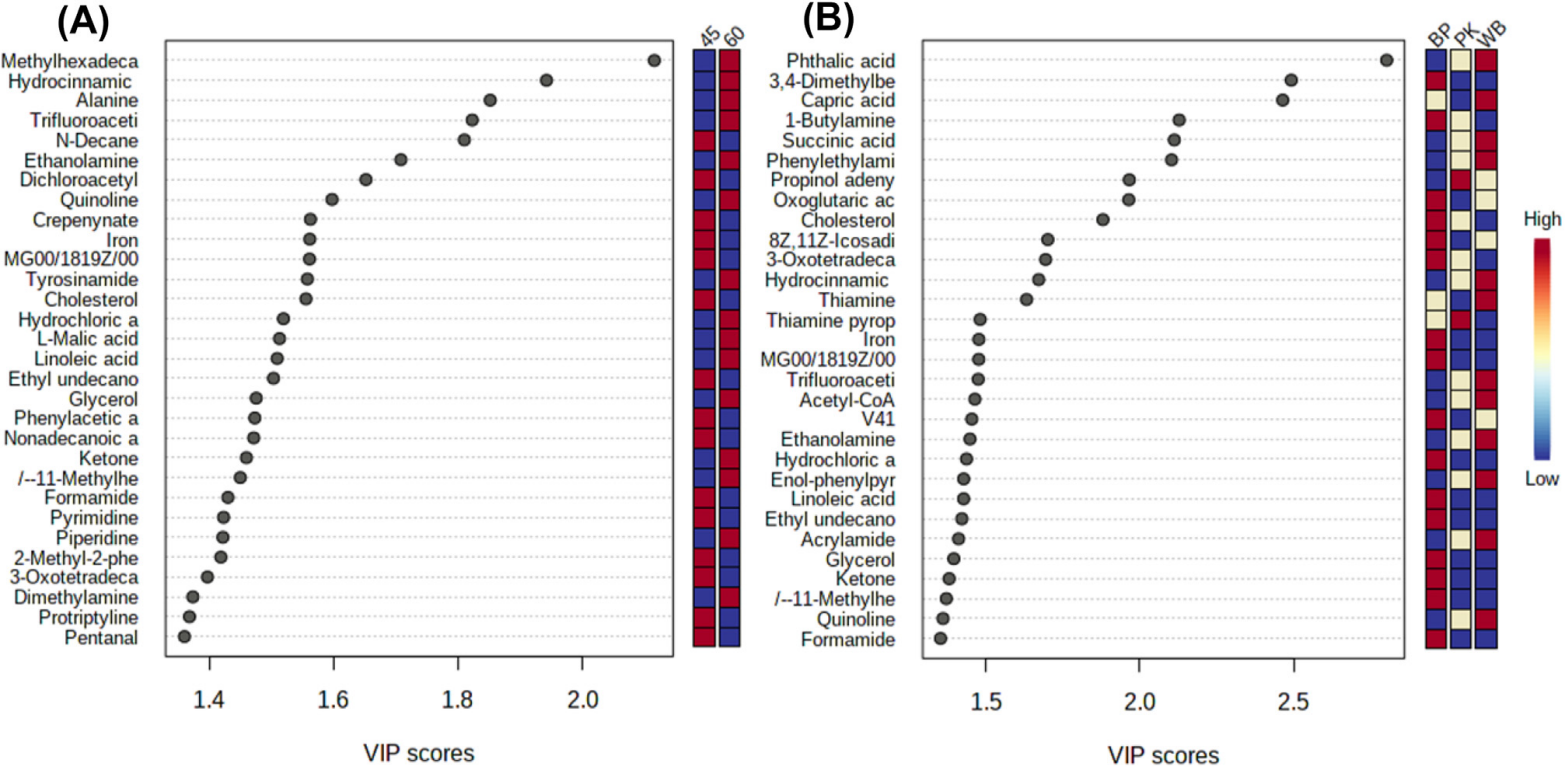
Top 30 significant compounds. Metabolites accountable for class discrimination with VIP >1 among crude fiber levels (a; 6% vs. 4.5%) and fiber sources (b; BP, PK, and WB) in 54 gestating sows. BP = beet pulp; PK = palm kernel meal; WB = wheat bran.

**Fig. 8:**
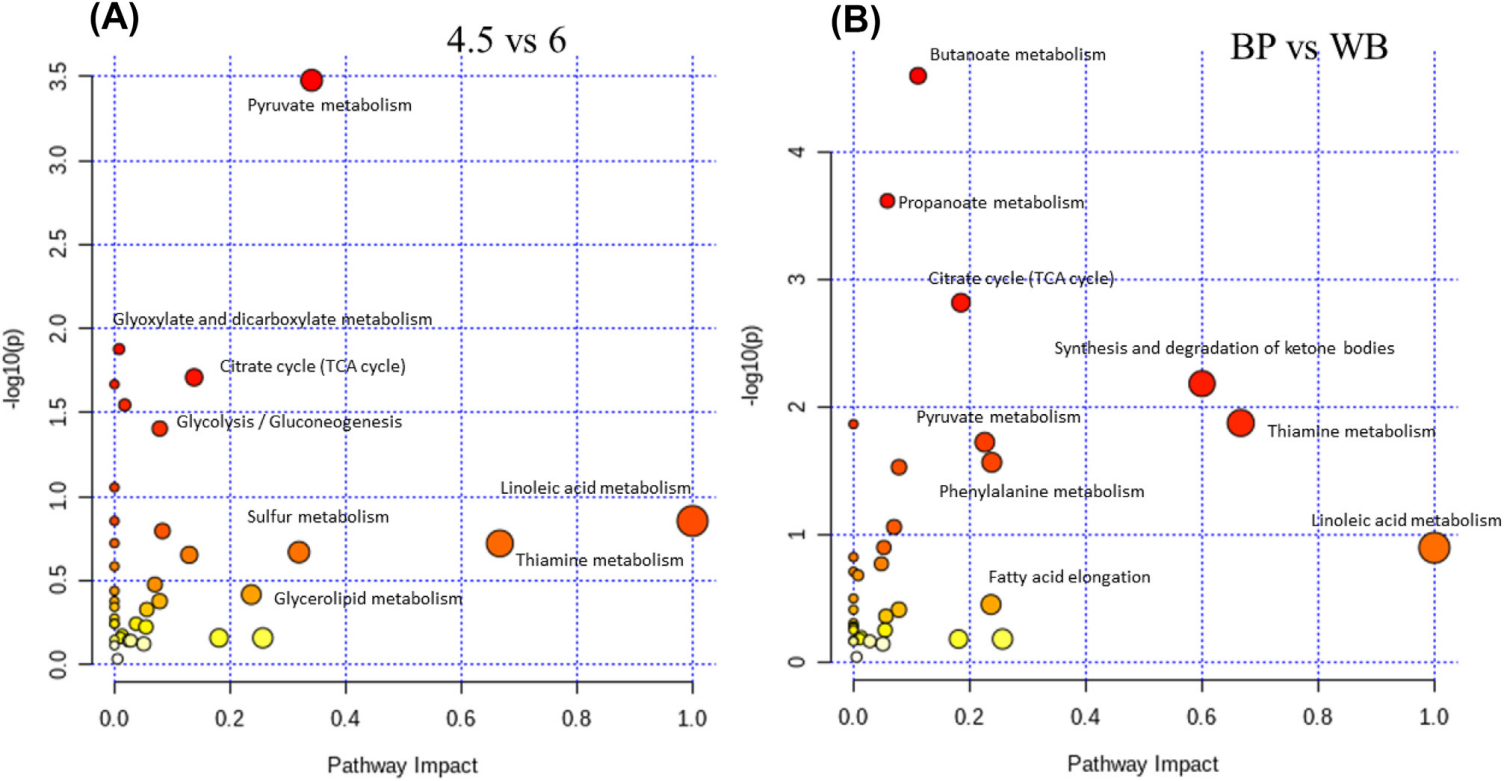
Metabolome view map of the differential metabolites (VIP >1, *P* < 0.05) identified in the feces of sows fed different crude fiber levels (a; 4.5% vs. 6%) and fiber sources (b; BP vs. WB) during late gestation (54 sows). The x-axis represents the pathway impact and the y-axis represents the pathway enrichment. The node color is based on its *P*-value, and the node radius is determined based on the pathway impact values. Larger sizes and darker colors represent higher pathway enrichment and impact values, respectively. BP = beet pulp; WB = wheat bran.

The parametric or non-parametric analyses showed that there were significant differences in the distribution of 30 metabolites among fiber level (Fig. 7a) and fiber source (Fig. 7b) treatments (*P* < 0.05). The VIP value of 66 metabolites was greater than 1.0 in different fiber levels. The VIP scores of methylhexadecanoic acid, hydrocinnamic acid, alanine, trifluoroacetic acid, ethanolamine, quinoline, tyrosinamide, hydrochloric acid, L-malic acid, linoleic acid, glycerol, ketone, 11-methylhexadecanoic acid, piperidine, and dimethylamine were greater (*P* < 0.05) in sows fed with 6% CF, whereas the VIP scores of n-decane, dichloroacetyl chloride, crepenynate, iron, cholesterol, ethyl undecanoate, phenylacetic acid, nonadecanoic acid, formamide, pyrimidine, 2-methyl-2-phenyl-undecane, 3-oxotetradecanoyl-acp, protriptyline, and pentanal were increased (*P* < 0.05) in sows fed with 4.5% CF. The concentration of phthalic acid, succinic acid, phenylethylamine, hydrocinnamic acid, trifluoroacetic acid, acetyl-CoA, ethanolamine, enol-phenylpyruvate, acrylamide, and quinoline were increased (*P* < 0.05) in the WB treatment compared with the BP, whereas 3,4-dimethylbenzoic acid, 1-butylamine, cholesterol, 3-oxo tetradecanoic acid, iron, hydrochloric acid, linoleic acid, ethyl undecanoate, glycerol, ketone, 11-methylhexane, and formamide were increased (*P* < 0.05) in the BP treatment compared with the WB. Greater (*P* < 0.05) capric acid and thiamine concentrations have been observed in fecal samples in the WB treatment compared with the PK; however, thiamine pyrophosphate concentration was increased (*P* < 0.05) in the PK treatment. The BP treatment showed a greater (*P* < 0.05) 3,4-dimethylbenzoic acid, oxoglutaric acid, 8z,11z-icosadienoyl-coa, iron, v41, hydrochloric acid, linoleic acid, ethyl undecanoate, glycerol, ketone, 11-methylhexane, and formamide concentrations in the feces rather than the PK treatment, whereas a greater (*P* < 0.05) propanol adenylate level was observed in the PK treatment. The metabolic pathways analysis showed that the high fiber diets affected (*P* < 0.05) the linoleic acid, thiamine, and pyruvate metabolism compared with the low fiber treatments (Fig. 8a). The comparison between the BP and WB treatments showed the change in linoleic acid, thiamine metabolisms, and synthesis and degradation of ketone bodies (Fig. 8b).

## 4 Discussion

The insignificant effect of fiber source and level on body weight change is in agreement with previous studies (Jørgensen et al., 2007; Li et al., 2021; Mota-Rojas et al., 2021). Limited studies have investigated the effect of fiber level and types in gestating sows subjected to heat stress; however, pregnant sows may have different requirements regarding the extent of environmental stressors and fetus physiological requirements. Using high fiber level in the diet is a common strategy to improve gestating sow productivity (Meunier-Salaün et al., 2001). To the best of the authors knowledge, the current study is the first to evaluate the metabolic and physiological effects of different fiber levels and sources on the reproductive performance of sows during heat stress. The effects of dietary fiber on intestinal motility and the risk of constipation before farrowing were previously reported by Oliviero et al. (2009). The fecal score also has a positive correlation with dietary fiber in gestating sows (Wu et al., 2021a). However, the reported results are based on the normal environment and the use of high fiber during heat stress is mostly ignored in order to reduce intestinal motility and heat increment. Our results suggest positive roles of dietary SF in high ambient temperatures, as indicated by the improved constipation index and shorter farrowing duration. The relationship between shorter farrowing duration and greater piglet survivability has already been established in several farm-based experiments (Choi et al., 2018; Kim et al., 2019; Liu et al., 2021; Thorsen et al., 2017). Farrowing duration appears to be a determinant factor in sows' reproductive performance since it physiologically affects piglets and sows (Choi et al., 2017; Thorsen et al., 2017). Prolonged labor is a painful period and induces stress on sows and piglets, thereby increasing the number of stillbirths (Thorsen et al., 2017). As the duration of farrowing exceeds 4 h, the rate of piglet mortality increases (Jiang et al., 2019; Lee et al., 2019). In this study, the high average farrowing duration (4.65 h) may be due to the adverse influences of heat stress. The beneficial effects of high SF diets on farrowing duration may be due to the increased constipation index. Gestating sows, in particular, are at risk of constipation due to a restricted diet from 2 d before the expected parturition (Choi et al., 2019; Oliviero et al., 2009). Constipation adversely affects sows health by increasing distress symptoms, gut obstruction, and prolonged farrowing duration (Thorsen et al., 2017). Acknowledging the effects of dietary fiber, enriching the diet with SF in the transition period to reduce farrowing duration can be used as a procedure to enhance sow productivity during heat stress.

In the current study, litter growth was not affected by fiber level; however, sows fed BP had a greater litter performance compared with sows fed PK or WB. The increased level of dietary SF improved litter performance due to higher SCFA production (Jiang et al., 2019; Li et al., 2021; Oliviero et al., 2009). The production of SCFA decreased the incidence of constipation and improved intestinal integrity, leading to increased health and welfare of gestating sows (Gu et al., 2021; He et al., 2019; Oliviero et al., 2009; Taciak et al., 2010). Healthier sows may have improved intestinal integrity and greater feed intake, which ensure mammary gland development and milk production. A considerable rate of mammary gland development occurs during the last weeks of gestation from d 80 afterward (Farmer et al., 2015; Tian et al., 2018). The secretion of endocrine hormones at the last third of gestation significantly improves the development of epithelial tissues in the alveoli (Farmer et al., 2015; He et al., 2019; Lin et al., 2016). However, the majority of previous studies showed no litter performance differences using SF and ISF at late gestation or during lactation in normal environmental temperatures (Yan et al., 2020). Therefore, the SF improves the constipation index and fecal texture, which consequently improves the digestion of nutrients.

The treatments had no effect on the digestibility of DM and GE; however, the digestibility of CP was improved in the BP treatment. The soluble fraction of fibers is composed of hemicellulose and pectin, which can be fermented by intestinal microbiota (Li et al., 2021; Lindberg, 2014; Mota-Rojas et al., 2021). Therefore, the SF improves the constipation index and fecal texture, consequently improving the digestion of nutrients. However, the digestibility of ADF was not improved in the BP treatment, showing that the insoluble fraction of fibers is not under influence of fermentation and remains undigested. Interestingly, despite the insignificant difference in ADF digestibility, there was an interaction for improved NDF digestibility between BP supplementation and a low fiber diet (4.5%).

Soluble fibers were considered to have acetate-producing properties rather than butyrate production (Bench et al., 2013; Li et al., 2021; Taciak et al., 2010). A high dietary fiber during late gestation enhanced SCFA production at the distal part of the intestine due to relatively higher SF fermentation (Taciak et al., 2010). Several physiological activities, including lipid metabolism, carbohydrate homeostasis, immunity, and intestinal integrity, can be modulated by SCFA production (Chen et al., 2021; Jiang et al., 2019; Lindberg, 2014). The body metabolism regulation occurs by SCFA effects on histone deacetylation control through binding with G-protein-coupled receptor 41 and G-protein-coupled receptor 43 in the intestine to restrict histone deacetylation and have a positive role in body metabolism (Tolhurst et al., 2012). The pattern of fermentation and acetate production in the colon has been suggested to be important in reducing anti-inflammatory metabolites (Fukuda et al., 2011) leading to gut health and growth. Moreover, the production of SCFA improves barrier function and reduces permeability in intestinal epithelial cells (He et al., 2019), which is a determinant factor in modulating intestinal health, nutrient digestibility, and inflammatory responses. Our previous study showed that the ISF can also have a positive role in reducing constipation and increasing intestinal health due to high water holding capacity (Jørgensen et al., 2007). Moreover, some carbohydrates can be encompassed in fiber structures and escape the digestion process in the small intestine, which is thought to provide further fermentation substrates in distal sections (Hosseindoust et al., 2019). The current study highlights the importance of SF as substrates for fermentation and SCFA production.

Cortisol secretion is a common physiological reaction during a stressful period (Nejad et al., 2019). The results of our study identified that an increase in hair cortisol is associated with an increase in ambient temperature. The response to cortisol secretion during heat stress is strong in female animals due to fetus growth, metabolic status, and immunological change (Choi et al., 2018). Moreover, our results showed that the respiratory rate was reduced in sows fed a high fiber diet, indicating lower stress. The reduced respiratory rate was in connection with the lower hair cortisol concentration in the high fiber treatment. Jiang et al. (2019) reported decreasing blood cortisol concentration when the dietary fiber level increased. Previous investigations have confirmed that hair cortisol is a more suitable indicator of chronic heat stress than blood cortisol due to the long-term deposition of cortisol in the hair. The addition of 7.5% CF to the diet reduced the cortisol content in feces and saliva compared with gestating sows fed 2.5% dietary CF (Jiang et al., 2019). Hair cortisol is not well studied in gestating sows, but the correlation between chronic stress and hair or wool cortisol has been confirmed in cattle (Ghassemi Nejad et al., 2017) and sheep (Ghassemi Nejad et al., 2014, 2020). The mechanisms underlying the effects of fiber source or level on stress factors in transitioning sows have not been confirmed, but the feeling of satiety may be related to lower stress.

The increase in the lying posture and decrease in standing and sham chewing behaviors in the BP and high fiber treatments were associated with a reduced respiratory rate. The increased lying behavior indicates comfort (He et al., 2019; Mós et al., 2020) and sham chewing shows stereotypical behavior in sows (Bench et al., 2013). In agreement, sows fed sugar beet pulp spent a lower time for standing behavior compared with sows fed potato pulp and pectin residue (Jørgensen et al., 2010). In this study, sows treated with BP had longer average durations of resting posture and shorter average durations of standing posture, suggesting that SF might enhance comfort during heat stress. Another explanation for the improved behavior is that SF increases SCFA production (Jørgensen et al., 2010), which increases intestinal peristalsis and constipation index to decrease the occurrence of gastrointestinal disorders. One potential reason for the improved behavioral factors in the high dietary fiber treatment might be the feeling of satiety. According to (Jiang et al., 2019), gestating sows provided a meal containing 7.5% CF displayed less stereotypical behavior and spent less time lying down compared to sows fed a diet containing 2.5% CF. This suggests that high dietary fiber inclusion can lower stress levels. Meunier-Salaün et al. (2001) reported that feed limitation increases stereotypical behavior such as sham chewing in sows. However, high dietary fiber has been advised under normal circumstances, and the role of dietary fiber during heat stress has rarely been studied (Chen et al., 2021). Our study results emphasize the necessity of including SF and ISF in the diet not only under normal conditions but also during high ambient temperatures to increase satiety and enhance the welfare of gestating sows. Therefore, the combination of SF and ISF may improve the behavioral factors and comfort in sows.

Recently, the role of dietary fiber in the regulation of metabolic pathways is receiving increasing attention. Although metabolite changes in response to heat stress are not well understood, general relationships between stressors and metabolite production have been reported in the literature. High-fiber diets reduce gut transit time and increase fermentation and production of SCFA such as acetate, propionate, and butyrate (Chen et al., 2021; Serena et al., 2009). Studies on the supplementation of SF and ISF indicated positive impacts of SF on SCFA production rather than protein-originated metabolites such as ammonia, phenol compounds, indole, cresol, and hydrogen sulfide (Serena et al., 2009). Phenylalanine, thiamine, pyruvate, and citrate cycle metabolisms were higher in the WB treatment compared with the BP. Amino acid pathways in the feces were enriched in high fiber diets compared to those in low fiber diets, although amino acids altered in feces do not have a direct one-to-one correspondence. Therefore, we hypothesized that the fecal enrichment of amino acids, pyruvate, and citrate cycle metabolisms observed in low fiber treatments may be correlated with intestinal damage. The altered metabolites in the high dietary fiber treatment were closely correlated with immunity. First, two essential amino acids, thiamine and phenylalanine, can regulate the immune system and hepatic performance, although there is a controversial result due to differences in the diets of animals and experimental models (Yan et al., 2020). It has been reported that alterations in amino acid metabolisms in the blood are negatively correlated with the fecal samples (Yan et al., 2020). The increased phthalic acid content in the feces of the WB sows was notable because phthalic acid is indicative of improved intestinal health and immunity (Wu et al., 2021b). 1-Butylamine, which possesses an ammonia-like odor (Bhuvaneswari et al., 2019), may be an indicator of undigested protein. The greater fecal 1-butylamine in sows fed the WB may be related to a decreased protein digestibility, leading to the increase of undigested protein flow to the colon.

## 5 Conclusion

In conclusion, the current study demonstrated that BP, a form of SF, improves the constipation index, farrowing duration, litter weight at weaning, digestibility of CP and NDF, acetate production, fecal metabolites, and behavioral factors of sows under heat stress. Furthermore, high dietary fiber increased sows behaviors associated with comfort, such as lying and sitting, and reduced stress indicators including respiratory rate and rectal temperature. Therefore, it appears necessary to add fiber to the diet of heat-stressed gestating sows, with a preference for using BP as a source of SF.

## Author contributions

**Seung Min Oh:** Formal analysis, Investigation, Visualization, and Project Administration. **Abdolreza Hosseindoust:** Conceptualization, Methodology, Writing Original Draft. **Sang Hun Ha:** Methodology. **Jun Young Mun:** Conceptualization. **Joseph Moturi:** Conceptualization. **Habeeb Tajudeen:** Validation. **Yo** **Han Choi:** Resources. **Su Hyup Lee:** Methodology. **Jin Soo Kim:** Conceptualization, Resources, Supervision, Project Administration, and Funding acquisition.

## Declaration of competing interest

We declare that we have no financial and personal relationships with other people or organizations that can inappropriately influence our work, and there is no professional or other personal interest of any nature or kind in any product, service and/or company that could be construed as influencing the content of this paper.
